# Langat virus infection affects hippocampal neuron morphology and function in mice without disease signs

**DOI:** 10.1186/s12974-020-01951-w

**Published:** 2020-09-20

**Authors:** Angela D. A. Cornelius, Shirin Hosseini, Sarah Schreier, David Fritzsch, Loreen Weichert, Kristin Michaelsen-Preusse, Markus Fendt, Andrea Kröger

**Affiliations:** 1 Innate Immunity and Infection, Helmholtz Centre for Infection Research, 38124 Braunschweig, Germany; 2 Present Address: Institute of Virology, Hannover Medical School, 30625 Hannover, Germany; 3 Department of Cellular Neurobiology, Zoological Institute, TU Braunschweig, 38106 Braunschweig, Germany; 4 Neuroinflammation and Neurodegeneration Group, Helmholtz Centre for Infection Research, 38124 Braunschweig, Germany; 5 Institute of Medical Microbiology and Hospital Hygiene, Otto-von-Guericke University, Leipziger Strasse 44, D-39120 Magdeburg, Germany; 6 Institute for Pharmacology and Toxicology, Otto-von-Guericke University, 39120 Magdeburg, Germany; 7 Center of Behavioral Brain Sciences, Otto-von-Guericke University, 39120 Magdeburg, Germany; 8 Gesundheitscampus Immunologie, Infektiologie und Inflammation (GCI3), Medical Center, Otto-von-Guericke University, 39120 Magdeburg, Germany

**Keywords:** Tick-borne encephalitis virus, Langat virus, Type I interferon, Inapparent infection, Hippocampus, Learning and memory

## Abstract

**Background:**

Tick-borne encephalitis virus (TBEV) is an important human pathogen that can cause the serious illness tick-borne encephalitis (TBE). Patients with clinical symptoms can suffer from severe meningoencephalitis with sequelae that include cognitive disorders and paralysis. While less than 30% of patients with clinical symptoms develop meningoencephalitis, the number of seropositive individuals in some regions indicates a much higher prevalence of TBEV infections, either with no or subclinical symptoms. The functional relevance of these subclinical TBEV infections and their influence on brain functions, such as learning and memory, has not been investigated so far.

**Methods:**

To compare the effect of low and high viral replication in the brain, wildtype and *Irf-7*^*−/−*^ mice were infected with Langat virus (LGTV), which belongs to the TBEV-serogroup. The viral burden was analyzed in the olfactory bulb and the hippocampus. Open field, elevated plus maze, and Morris water maze experiments were performed to determine the impact on anxiety-like behavior, learning, and memory formation. Spine density of hippocampal neurons and activation of microglia and astrocytes were analyzed.

**Results:**

In contrast to susceptible *Irf-7*^*−/−*^ mice, wildtype mice showed no disease signs upon LGTV infection. Detection of viral RNA in the olfactory bulb revealed CNS infections in wildtype and *Irf-7*^*−/−*^ mice. Very low levels of viral replication were detectable in the hippocampus of wildtype mice. Although wildtype mice develop no disease signs, they showed reduced anxiety-like behavior and impaired memory formation, whereas *Irf-7*^*−/−*^ mice were not affected. This impairment was associated with a significant decrease in spine density of neurons in the hippocampal CA1 region of wildtype mice. Microglia activation and astrogliosis were detected in the hippocampus.

**Conclusion:**

In this study, we demonstrate that subclinical infections by viruses from the TBEV-serogroup affected anxiety-like behavior. Virus replication in the olfactory bulb induced far-reaching effects on hippocampal neuron morphology and impaired hippocampus-dependent learning and memory formation.

## Background

Tick-borne encephalitis virus (TBEV) is a member of the genus *Flavivirus* in the family of *Flaviviridae*. Flaviviruses comprise many human pathogens, including Dengue virus (DENV), Japanese encephalitis virus (JEV), West Nile virus (WNV), Yellow fever virus (YFV), and Zika virus (ZIKV) [1].

TBEV is transmitted to humans primarily by infected ticks but can also be transmitted by dairy products from unpasteurized milk produced from infected livestock [2, 3]. Infection with TBEV causes tick-borne encephalitis (TBE) which affects the central nervous system (CNS). TBE is a typically biphasic disease. In the first phase of viremia, the dominant symptoms are fever, fatigue, general malaise, headache, and pain followed by a second phase with a clinical spectrum that ranges from mild meningitis to severe encephalitis with or without myelitis and spinal paralysis. More than 30% of patients with clinical symptoms caused by a TBEV infection develop prolonged sequelae that include neuropsychiatric symptoms, severe headaches, and a general decrease in quality of life [4, 5]. The total number of cases worldwide has been estimated to be up to 13,000 per year, and as such, the infection constitutes the most important tick-borne viral disease. Seroconversion without prominent morbidity is common. In two-thirds of infected humans, the infection is inapparent and without any disease signs [5–7]. Thus, possible cognitive impairments and impact on the behavior of patients with unrecognized infections are unknown.

To prevent TBEV infection, the Langat virus (LGTV), a member of the TBEV serogroup, was used as a vaccine against TBEV. In humans, LGTV is a low pathogenic flavivirus, and it shares 82–88% amino acid homology with TBEV. Vaccination with LGTV resulted in a neurological disease, with an incidence of approximately 1:18,500 and caused permanent neurologic sequelae among the vaccinated. In adult mice, LGTV generally has a low pathogenicity when inoculated subcutaneously or intracerebrally but is still associated with low-level viral replication in the brain [8].

The innate immune system is essential for the control of TBEV and LGTV infection. The type I interferon (IFN) system plays a pivotal role in the antiviral response against TBEV infections. The loss of type I IFN responses leads to higher viral replication and death of infected mice [8]. Interferon regulatory factor-7 (IRF-7) is a master regulator of type I IFN production, and *Irf-7*^*−/−*^ mice are highly susceptible to various viruses [9].

Activation of the innate immune system induces production of cytokines including interleukin-1β (IL-1β), IL-6, and tumor necrosis factor-α (TNF-α) and IFNs in the brain where they can have deleterious effects on cognitive and emotional behavior [10–13]. The hippocampus, which is essential for spatial learning and contextual memory formation, receives input from the entorhinal cortex, which relays through the dentate gyrus (DG) and the Cornu Ammonis regions CA1 and CA3. Inflammatory cytokines can negatively affect long-term potentiation (LTP) [14, 15], neuronal survival, synaptic plasticity, and memory formation [16–19]. It was shown that acute viral or bacterial infections of the CNS or the periphery could lead to changes in the neuronal complexity and functional deficits in hippocampal-dependent learning and memory [20, 21].

Whereas severe TBEV infection has long-term influence on behavioral function [6], the question arises, whether also inapparent infections of the CNS alter neural circuits and lead to learning and behavioral deficits. The aim of this study was to characterize the impact of low-pathogenic flavivirus infection, caused by LGTV in wildtype mice, on behavior and spatial memory. We compared findings with *Irf-7*^*−/−*^ mice, which showed high viral replication in the brain and weight loss but recovered from an infection. We found that inapparent infections lead to alterations in hippocampal structure and function and are associated with impairments in spatial learning in infected mice. Our findings indicate that non-recognized infections with neurotropic viruses can cause neurological changes.

## Material and methods

### Mice

All animal experiments were performed in compliance with the relevant animal welfare law (EU-Directive 2010/63/EU). The mice were housed and handled in accordance with good animal practice as defined by FELASA. All animal experiments were approved by the Lower Saxony State Office of Consumer Protection and Food Safety under permit number AZ 33.9-42502-04-15/1950 or by the Landesverwaltungsamt Sachsen-Anhalt AZ 42502-2-1344, University Magdeburg. Wildtype C57BL/6 J (WT) and *Irf-7*^*−/−*^ mice were bred under SPF conditions at the Helmholtz Centre for Infection Research in Braunschweig or at the Otto-von-Guericke University Magdeburg, Germany. To exclude gender effects, all experiments were performed with male and female mice. Six- to 10-week-old mice were infected intraperitoneally (i.p.) with 10^4^ focus-forming units (FFU) LGTV in 100 μl PBS or mock injected with 100 μl PBS. Mice that lost 20% or more of their body weight or showed pain were sacrificed. For analysis, all mice were killed by transcardial perfusion with 30 ml PBS during anesthesia (100 μl/10 g body weight ketamine (150 mg)/xylazine (9 mg)). For histological analysis, PBS perfusion was followed by perfusion with 30 ml 4% paraformaldehyde.

### Virus

LGTV strain TP21 (G. Dobler) was propagated in Vero B4 cells. Supernatants were cleared for cell debris by centrifugation. Titers were determined by focus-forming assays on Vero B4 cells [22].

### RNA extraction and real-time RT-PCR

For RNA extraction, mouse organs were homogenized in peqGOLD TriFast (PeqLab) using a Fast Prep 24 homogenizer (MP Biomedicals). RNA was isolated according to the manufacturer’s instructions. cDNA synthesis was performed using M-MLV Reverse Transcriptase Kit (Invitrogen/Life technologies). RNA was quantified with the KAPA probe FAST qPCR kit using primers amplifying LGTV NS3 (forward primer 5′-AAC GGA GCC ATA GCC AGT GA-3′, reverse primer 5′-AAC CCG TCC CGC CAC TC-3′, probe FAM-AGA GAC AGA TCC CTG ATG G-MGB), IL-6 (forward primer 5′-AGT TGC CTT CTT GGG ACT GA-3′, reverse primer 5′-CAG AAT TGC CAT TGC ACA AC-3′), TNF-α (forward primer 5′-GAA CTG GCA GAA GAG GCA CT-3′, reverse primer 5′-AGG GTC TGG GCC ATA GAA CT-3′), and β-actin (forward primer 5′-TGG AAT CCT GTG GCA TCC ATG AAA-3′, reverse primer 5′-TAA AAC GCA GCT CAG TAA CAG TCC G-3′). Samples were measured by a Light Cycler 480 II (Roche). The target/β-actin ratio was analyzed by the Light Cycler 480 II software (Roche).

### Brain immune cell analysis

Mouse brains were harvested from uninfected or LGTV-infected mice. Following perfusion, brains were homogenized through a 70-μm cell strainer into DMEM medium (Gibco® Life Technologies). Cells were separated by centrifugation on a discontinuous 30 to 70% percoll gradient. All samples were incubated with FcR blocking reagent (Miltenyi Biotec) for 10 min at 4 °C and immunolabeled with different antigen-specific fluorescent antibodies (anti-CD45 (30-F11), anti-CD11b (M1/70), anti-CD3 (145-2C11; BD Biosciences)). Brain leukocyte numbers were quantitated using TruCount beads (BD Biosciences). Analysis was performed on BD LSRII using the BD FACSDiva and FlowJo software.

### Immunohistochemistry

Immunohistological analyses were performed on mouse brains harvested after cardiac perfusion with 1x PBS followed by 4% PFA in 1x PBS. Brains were removed and fixed in 4% PFA in 1x PBS for 24 h and transferred into 30% sucrose (in 1x PBS) solution for 48 h. Fixed brains were frozen into Tissue-Tek® (Sakura) and stored at − 20 °C. Frozen and embedded brains were cut with a cryomicrotome (Leica) into 50-μm sections. Sections were placed on gelatin-coated microscope slides (protocol after R&D Systems®) and were incubated for 1 h at room temperature (RT) in blocking solution containing 0.2% Triton X-100, 1% BSA, and 10% goat serum in 1x PBS. Primary polyclonal antibodies anti-GFAP (guinea pig, Synaptic Systems), anti-IBA1 (guinea pig, Synaptic Systems), and monoclonal anti-TBEV E (antiserum 1786) [23], respectively, were added for 2 h at RT in 0.2% Triton X-100 and 10% goat serum in 1x PBS. After washing tissue sections twice for 5 min in 1x PBS, the secondary antibodies (goat anti guinea pig, IgG/H+L, Alexa Fluor® 647 and goat anti-mouse, IgG+IgM, Cy3 Jackson IRL) were added for 1 h at RT together with DAPI (1:1000) in 1x PBS. Sections were washed two times for 5 min in 1x PBS and mounted with Neo-Mount® (Sigma-Aldrich). Representative images were acquired with an LSM 710 (Zeiss) microscope at × 20 and × 40 magnifications.

### Behavioral experiment design

For behavioral evaluation, wildtype and *Irf-7*^*−/−*^ mice were assigned to different groups. All behavioral experiments were performed with the same cohort of LGTV infected (WT: *n* = 10; *Irf-7*^*−/−*^: *n* = 9) or uninfected WT (*n* = 9) and *Irf-7*^*−/−*^ (*n* = 7) mice at the same time of day during the light cycle under a dim light illumination between 9:00 to 16:00 o’clock by an experimenter blinded to all groupings.

In this study, both male and female mice within all the experimental groups exhibited similar characteristics without any significant differences allowing us to pool and present data obtained from animals of both genders together.

**Fig. 1:**
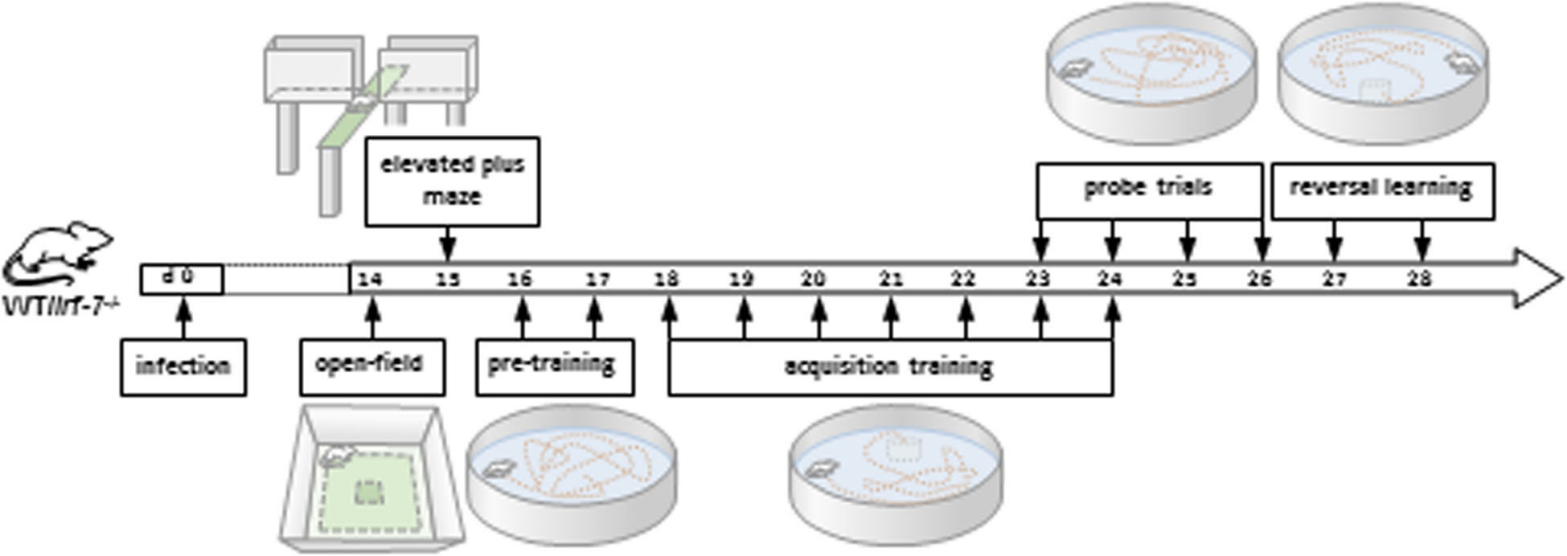
Schematic presentation of the experimental design

To ensure that mice of both genotypes showed no deficits from infection in locomotion and exploratory activity, open field and elevated plus maze test for anxiety assessment were performed from 14 days after LGTV infection. Twenty-four hours later, to investigate the cognitive behavior of the mice, spatial learning and memory formation was assessed using the Morris water maze paradigm. Prior to the acquisition, a visible platform task was performed as a pre-training for 3 consecutive days. Afterwards, the mice were tested for 9 days in initial learning Morris water maze (acquisition and probe trial). After another 24 h, the mice were tested for another 4 days (reversal acquisition and probe trial) in the reversal Morris water maze task (Fig. 1).

#### Open field test

The open field test was performed as described previously [24]. Briefly, mice were placed along one side of a white PVC open field apparatus (40 × 40 × 40 cm) for 5 min. The central area of the arena was specified as the center zone (30 × 30 cm), and the exact center of the arena was considered as the core zone (10 × 10 cm). Between each session of experiments, the apparatus was cleaned with Bacillol (Hartmann Bode). Movement data, including total distance traveled, average speed, and percentage of activity in the border and in the center zones of the arena were collected by the ANY-maze behavioral tracking software (Stoelting).

#### Elevated plus maze test

The elevated plus maze test was performed as described previously [25]. In this test, the apparatus was composed of a cross with two opposed open arms (25 × 5 cm) and two opposed closed arms (25 × 5 cm, surrounded by 20 cm high walls). The white PVC open field apparatus was elevated 50 cm above the floor. Mice were placed in the central part of the arena (5 × 5 cm) facing towards an open arm and permitted to move freely in the arena for 5 min. Locomotion data, including the percentage of time spent in open and closed arms, were collected by the ANY-maze behavioral tracking software (Stoelting).

#### Morris water maze test

Spatial learning was assessed using the initial training and the reversal learning phase of the Morris water maze (MWM) paradigm [26, 27]. The MWM test was performed as previously described [20]. In brief, a circular pool with a diameter of 150 cm with an escape platform of 10 cm in diameter located 1.0 cm below (hidden) the water surface was used. The temperature of the water was kept constant throughout the experiment (20 ± 0.5 °C), and a 10 min recovery period was allowed between the training trials. Pre-training was performed with a visible platform to ensure adequate swimming ability and visual acuity. Two trials (maximum of 60 s each) per day were performed for two consecutive days. Subsequently, training in the MWM test was performed for 8 days with a hidden platform located in the NE quadrant with variable starting points (SE, S, W, and NW). If the mouse failed to find the escape platform within 60 s, the mouse was guided to the platform and allowed to sit there for 20 s. The pathway map to find the platform was analyzed for: (i) searching strategies including scanning characterized by 10–60% surface coverage, (ii) changing characterized by > 80% time in a doughnut-shaped annulus zone, (iii) random swimming characterized by > 60% surface coverage of the whole pool area, and in (iv) a directed search characterized by > 80% time in Wishaw’s corridor [28–30].

For memory retrieval assessment, two reference memory tests (probe trials) were performed at the third and sixth day of the acquisition training, before starting the training trials. The third probe trial test was performed 24 h after the last day of acquisition training. During probe trials, the platform was removed, and the animals were allowed to swim freely for 45 s. To test the ability of the animals to form a new memory, the platform was moved to the opposite quadrant of the pool (SW) after the third probe trial test. The reversal learning task consisted of 3 training days. Data were collected by the ANY-maze behavioral tracking software (Stoelting).

### Golgi-Cox staining

Mice were killed by CO_2_ inhalation, and brains were immediately isolated. The right hemisphere was incubated in FD rapid Golgi stain kit (FD NeuroTechnologies) according to the manufacturer’s protocol. Afterwards, hemispheres were blocked in 2% agar, and 200-μm thick coronal sections were cut with a vibratome (Leica, VT 1000 S) and mounted on gelatin-coated glass slides. Subsequently, sections were processed for signal development before dehydration through graded alcohols and mounting using Permount (Thermo Fisher Scientific). Analysis of spine density was performed using ImageJ, counting dendritic spines in a defined length (> 60 μm) of a dendrite. In each mouse, 10 dendrites located in CA1 apical, basal, and dentate granule cells located in superior and inferior sub-regions of the hippocampus were selected and counted by an investigator blinded to all experimental groups.

### Statistical analysis

Data were analyzed and plotted using the GraphPad Prism 6.0 software (GraphPad Software Inc., La Jolla, CA, USA). All values are presented as means ± standard error of the means (SEM). One-way and two-way ANOVA, followed by post hoc Student’s *t* test using Fisher’s least significant differences, was employed to analyze the data. The minimum significance value was considered *p* < 0.05. All experiments were blindly analyzed.

## Results

### Characterization of LGTV infection

**Fig. 2:**
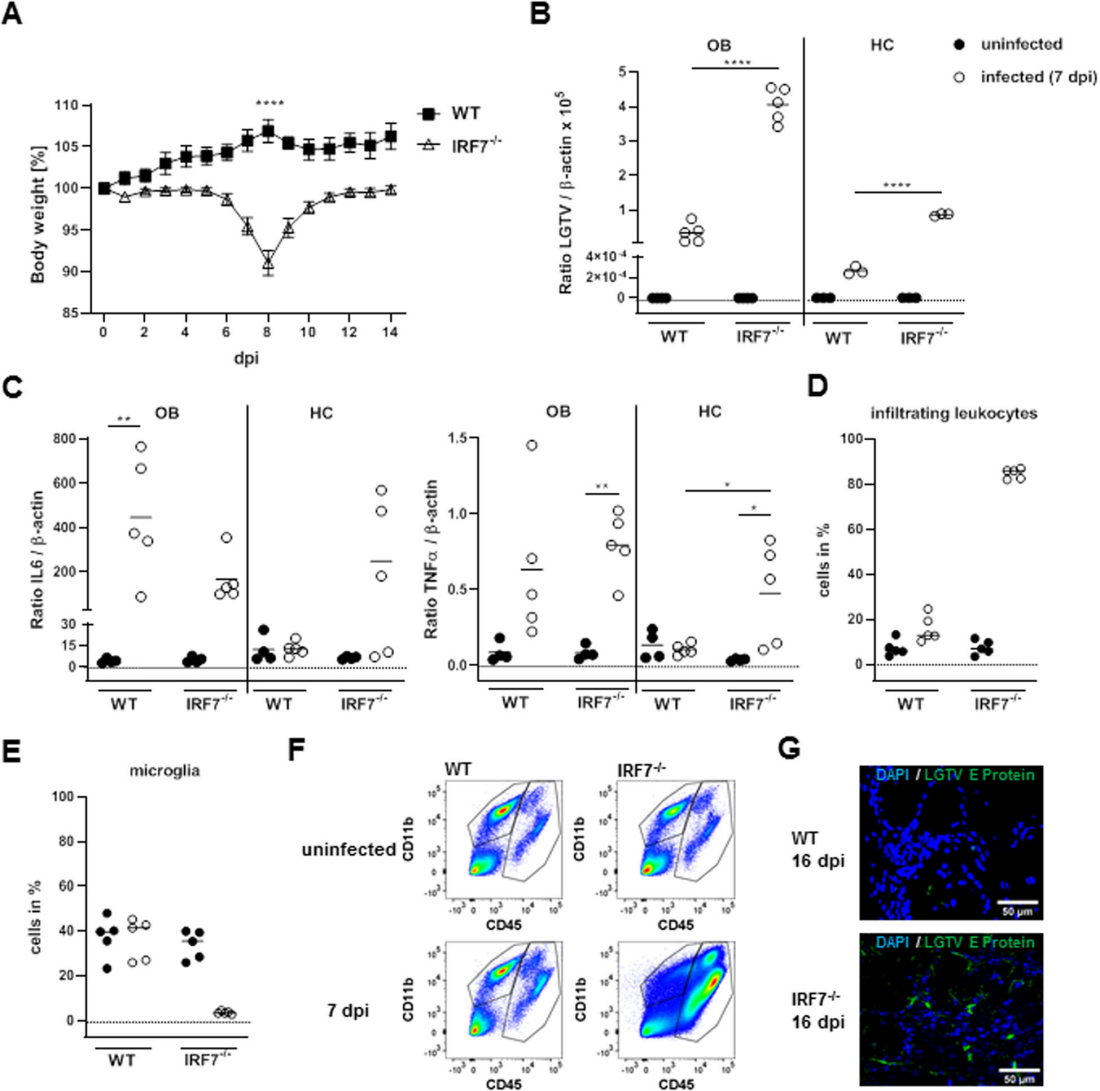
Langat virus infection leads to asymptomatic infection in wildtype mice and brain region specific replication. Wildtype (*n* = 8) and *Irf-7*^*−/−*^ mice (*n* = 10) were intraperitoneally infected with 10^4^ FFU of LGTV. **a** Body weight. Data represent the mean with SEM of *n* ≥ 8 mice per group and time point. Quantitative RT-PCR was performed for the olfactory bulb (OB) and the hippocampus (HC). Results were normalized to β-actin for **b** LGTV, and **c** IL-6 and TNF-α. The dotted line represents the linear detection limit. Data represent the mean with individual values of *n* ≥ 5 per group and time point. For flow cytometry analysis of the brain, percentage of infiltrating cells (CD45^+^) (**d**) and microglia (CD45^lo/int^CD11b^+^) (**e**). **f** Representative dot blots of CD45 and CD11b staining are depicted: microglia (CD CD45^lo/int^CD11b^+^, infiltrating cells (CD45^+^)). **g** Immunhistological analysis of the olfactory bulb 16 days post-infection. DAPI (blue) and LGTV E-protein (green). Magnification × 40. Data were analyzed by two-way ANOVA with Tukey’s multiple comparison tests. *p < 0.05, **p < 0.01, ****p < 0.0001

To characterize the pathogenicity of LGTV infection in mice, wildtype and *Irf-7*^*−/−*^ mice were infected intraperitoneally. Both wildtype and *Irf-7*^*−/−*^ mice survived the infection. Wildtype mice showed no signs of infection, and the body weight was constant over time. However, *Irf-7*^*−/−*^ mice showed a mild reduction in body weight at day 8 of infection (Fig. 2a). Analysis of viral replication in the CNS by qRT-PCR revealed very low level of viral replication in the olfactory bulb of wildtype mice on day 7 of infection (Fig. 2b). There was only very low viral replication detectable in the hippocampus of wildtype mice (Fig. 2c). An elevated amount of viral RNA was detected in the olfactory bulb and the hippocampus of *Irf-7*^*−/−*^ mice as compared to wildtype mice (Fig. 2b, c). We further analyzed the expression of the proinflammatory cytokines IL-6 and TNF-α by qRT-PCR. Cytokine expression was induced upon infection in WT and *Irf-7*^*−/−*^ in the olfactory bulb. In the hippocampus, cytokine expression was only detectable in *Irf-7*^*−/−*^ mice (Fig. 2c). To evaluate the effect of infection on cell activation and infiltration of immune cells, we performed flow cytometry. Infection with LGTV leads to a decrease of microglia cells in WT mice, whereas no obvious change was determined in the percentage of infiltrating immune cells (Fig. 2d, e, f). In contrast, *Irf-7*^*−/−*^ mice showed massive infiltration of lymphocytes and monocytes to the brain upon LGTV infection (Fig. 2d. e). Histological analyses of virus-infected cells in the olfactory bulb revealed that the virus was cleared in wildtype mice, whereas infected cells were still detectable in *Irf-7*^*−/−*^ mice 16 days post-infection (Fig. 2f). Thus, these data indicate that infection with LGTV leads to virus replication in the brain without signs of disease in WT mice, whereas *Irf-7*^*−/−*^ mice showed high viral replication and mild disease signs. Surprisingly, low viral replication in the hippocampus was not able to induce cytokine expression.

### Open field

**Fig. 3:**
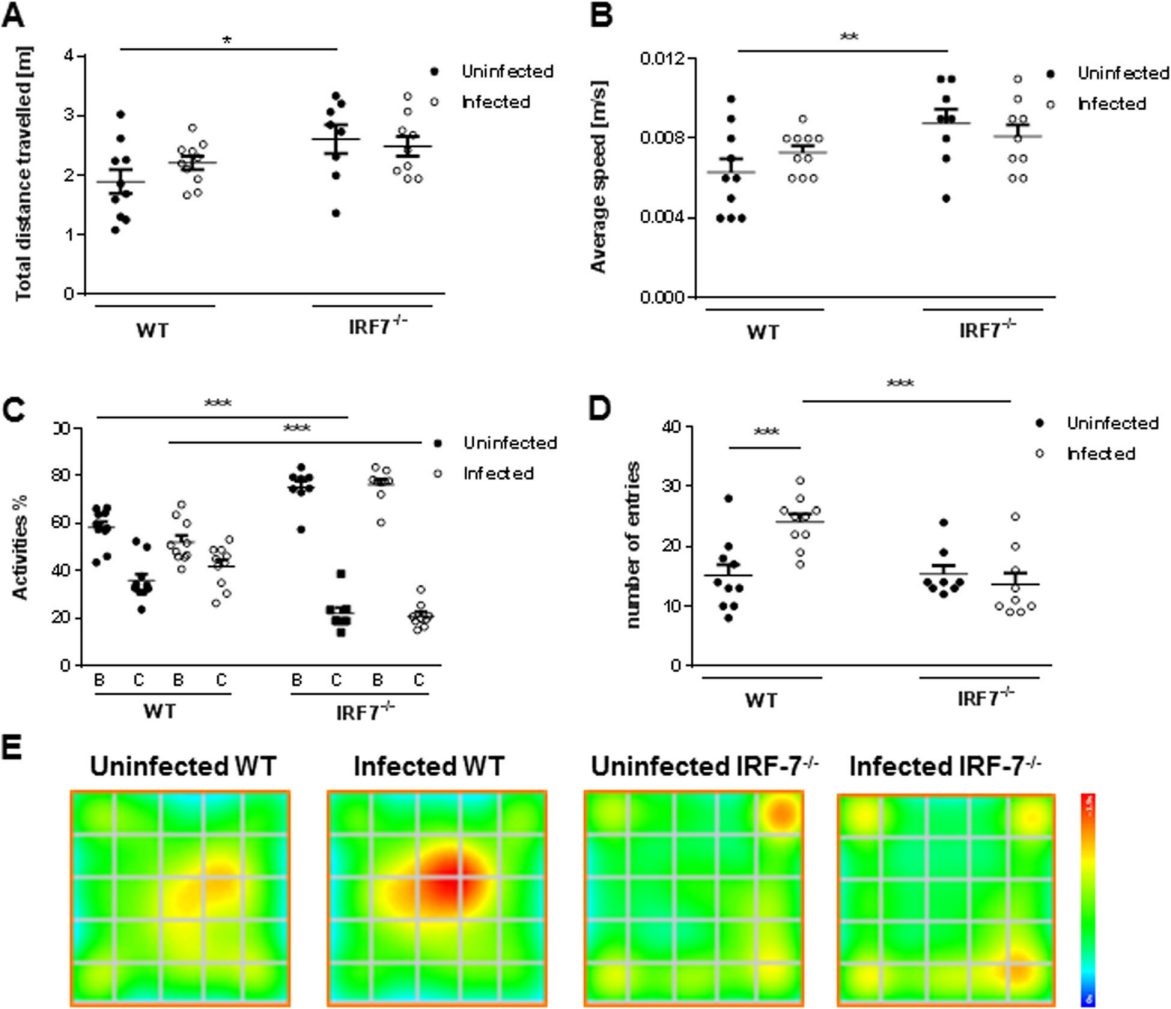
LGTV infection affected exploratory behavior in the open field test. Uninfected control (WT: *n* = 10, *Irf-7*^*−/−*^: *n* = 8) and LGTV-infected (10^4^ FFU, i.p.) mice (WT: *n* = 10, *Irf-7*^*−/−*^: *n* = 9) were tested for 5 min in the open field arena 14 days post-infection. **a** Total distance traveled, **b** average speed, **c** activity percentage of mice in the border (B) and central (C) zones of the open field arena, and **d** the number of core entries are presented. **e** Group mean heat map of the animals’ center position are illustrated for all tested groups. One-way ANOVA and post hoc Student’s *t* test using Fisher’s least significant differences were employed to analyze the data. Data are shown as mean ± SEM, *p < 0.05, **p < 0.01, and ***p < 0.001

To evaluate a potential effect of the LGTV infection on basal locomotor behavior (total distance traveled) and anxiety-related behavior (immobility, time spent in the center), the open field test was performed (Fig. 3). Total distance traveled and average speed were comparable between infected and non-infected in both wildtype and *Irf-7*^*−/−*^ mice (Fig. 3a, b) (total distance: WT d0 1.96 ± 0.20 m, WT d14 2.29 ± 0.11 m, *p* = 0.20; *Irf-7*^*−/−*^ d0 2.70 ± 0.24 m, *Irf-7*^*−/−*^ d14 2.58 ± 0.17 m, *p* = 0.66; average speed WT d0 0.006 ± 0.0007 m/s, WT d14 0.007 ± 0.0004 m/s, *p* = 0.19; *Irf-7*^*−/−*^ d0 0.009 ± 0.0008 m/s, *Irf-7*^*−/−*^ d14 0.008 ± 0.0006 m/s, *p* = 0.45). However, non-infected *Irf-7*^*−/−*^ mice in general showed an increased total distance traveled (*p* = 0.01) and average speed (*p* = 0.007) as compared to non-infected wildtype mice (Fig. 3a, b). The natural tendency of mice is to spend more time in the border zone of the open field arena. However, increased time spent in the border zone indicates anxiety-related behaviors. Therefore, to initially screen for anxiety-related behavior, the time spent in the border and the center zone of the arena was analyzed [31]. Although the time spent in the border zone of the open field arena did not change after infection in both wildtype and *Irf-7*^*−/−*^ mice (WT d0 58.33 ± 2.51 %, WT d14 52.19 ± 2.78 %, *p* = 0.09; *Irf-7*^*−/−*^ d0 75.11 ± 2.78 %, *Irf-7*^*−/−*^ d14 76.39 ± 2.26%, *p* = 0.74), it was higher in non-infected and infected *Irf-7*^*−/−*^ mice as compared to the respective wildtype mice (*p* < 0.001, Fig. 3c). However, infected wildtype mice showed significantly more core entries than uninfected wildtype mice, which was not the case for *Irf-7*^*−/−*^ mice (Fig. 3d) (WT d0 15.10 ± 1.87, WT d14 24.10 ± 1.32, *p* < 0.001); *Irf-7*^*−/−*^ d0 15.38 ± 1.44, *Irf-7*^*−/−*^ d14 13.67 ± 1.87, *p* = 0.49). Thus, the infection did not influence locomotor activity of mice, regardless of the genotype. In general, *Irf-7*^*−/−*^ mice showed increased anxiety-related behavior as compared to non-infected WT mice regardless of virus infection. Infected WT mice showed signs of reduced anxiety-related behavior as the number of core entries were increased as compared to uninfected mice (Fig. 3e).

### Elevated plus maze

**Fig. 4:**
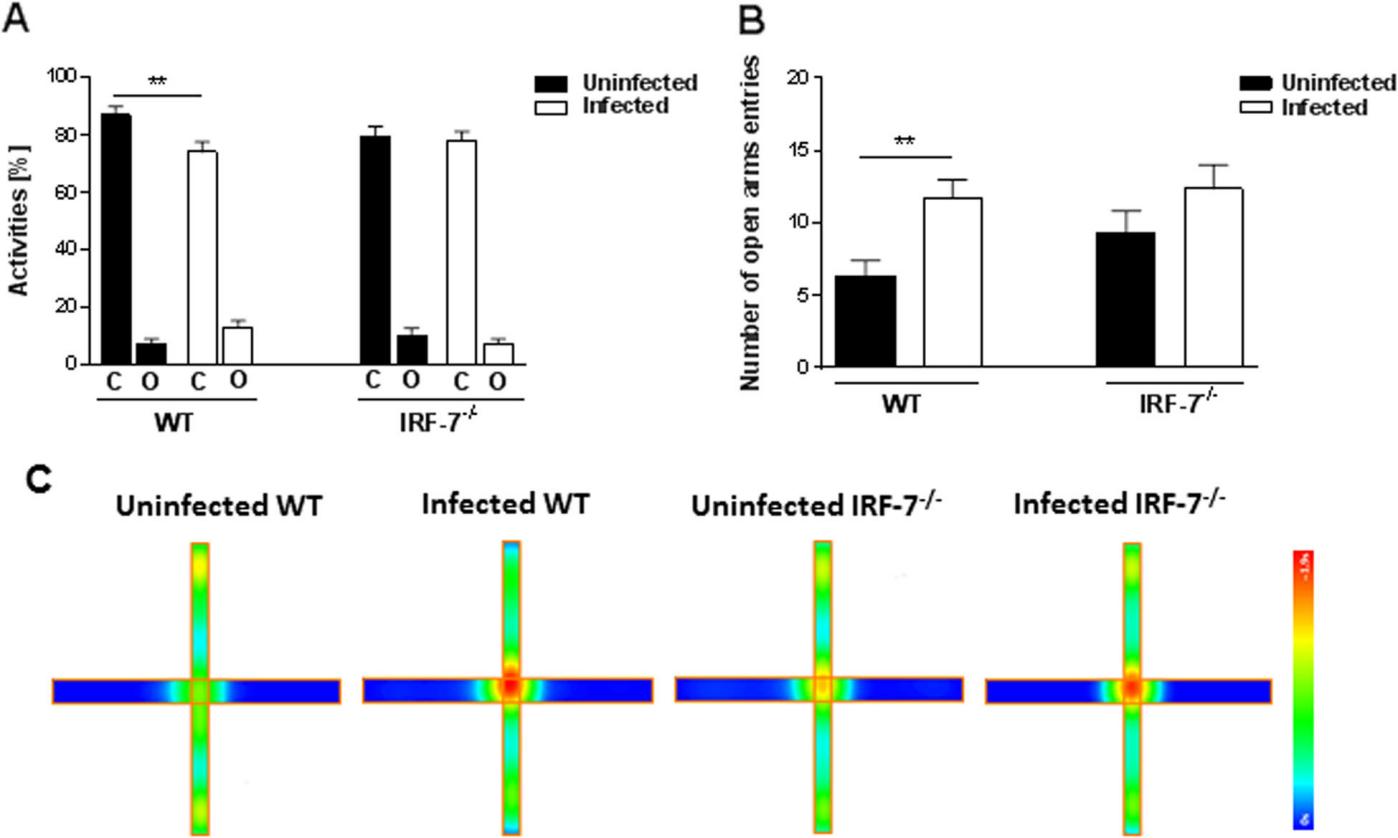
LGTV infection diminished anxiety-like behavior in wildtype mice. Uninfected control (WT: *n* = 10, *Irf-7*^*−/−*^: *n* = 8) and LGTV-infected (10^4^ FFU, i.p.) mice (WT: *n* = 10, *Irf-7*^*−/−*^: *n* = 9) were tested for 5 min in the elevated plus maze. **a** Activity percentage of mice in the closed arms (C) and open arms (O) of the elevated plus maze and **b** the number of entries to the open arms are shown. **c** Group mean heat maps of the animals’ center position are illustrated for all tested groups. One-way ANOVA and post hoc Student’s *t* test using Fisher’s least significant differences were employed to analyze the data. Data are shown as mean ± SEM, **p < 0.01

For a more detailed analysis on the impact of LGTV infection on anxiety behavior, the elevated plus maze test was performed (Fig. 4). In this test as well, the natural tendency of mice is to spend more time in the closed arms of the maze, however, an increase in closed arm activity (duration and/or entries) indicates anxiety-like behavior in rodents [32].

Although all tested animals spent more time in the closed arms than in the open arms of the elevated plus maze, LGTV infection in wildtype mice led to a significant reduction in the time spent in the closed arm (Fig. 4a) and increased numbers of entries to the open arms (Fig. 4b) compared to uninfected wildtype mice (time in closed arms: uninfected WT 87.06 ± 2.84 %, infected WT 74.02 ± 3.55%, *p* = 0.007; entries in open arms: uninfected WT 6.30 ± 1.11%, infected WT 11.70 ± 1.28%, *p* = 0.008). In contrast, LGTV infection of *Irf-7*^*−/−*^ mice had no impact on time spent in the closed arms (and number of entries in the open arms) compared to uninfected *Irf-7*^*−/−*^ mice (Fig. 4a–c) (time in closed arms uninfected *Irf-7*^*−/−*^ 79.24 ± 3.75, infected *Irf-7*^*−/−*^ 77.83 ± 3.40, *p* = 0.77; entries in open arms: uninfected *Irf-7*^*−/−*^ 9.25 ± 1.57, infected *Irf-7*^*−/−*^ 12.33 ± 1.66, *p* = 0.14). Overall, LGTV infection of wildtype mice diminished anxiety-like behavior.

### Morris water maze

To investigate the effects of LGTV infection on cognitive function, training in the Morris water maze task was performed. During 3 days of pre-training, swimming ability and visual acuity were intact in all animals as the swimming speed and escape latency were comparable in all tested groups (data are not shown).

**Fig. 5:**
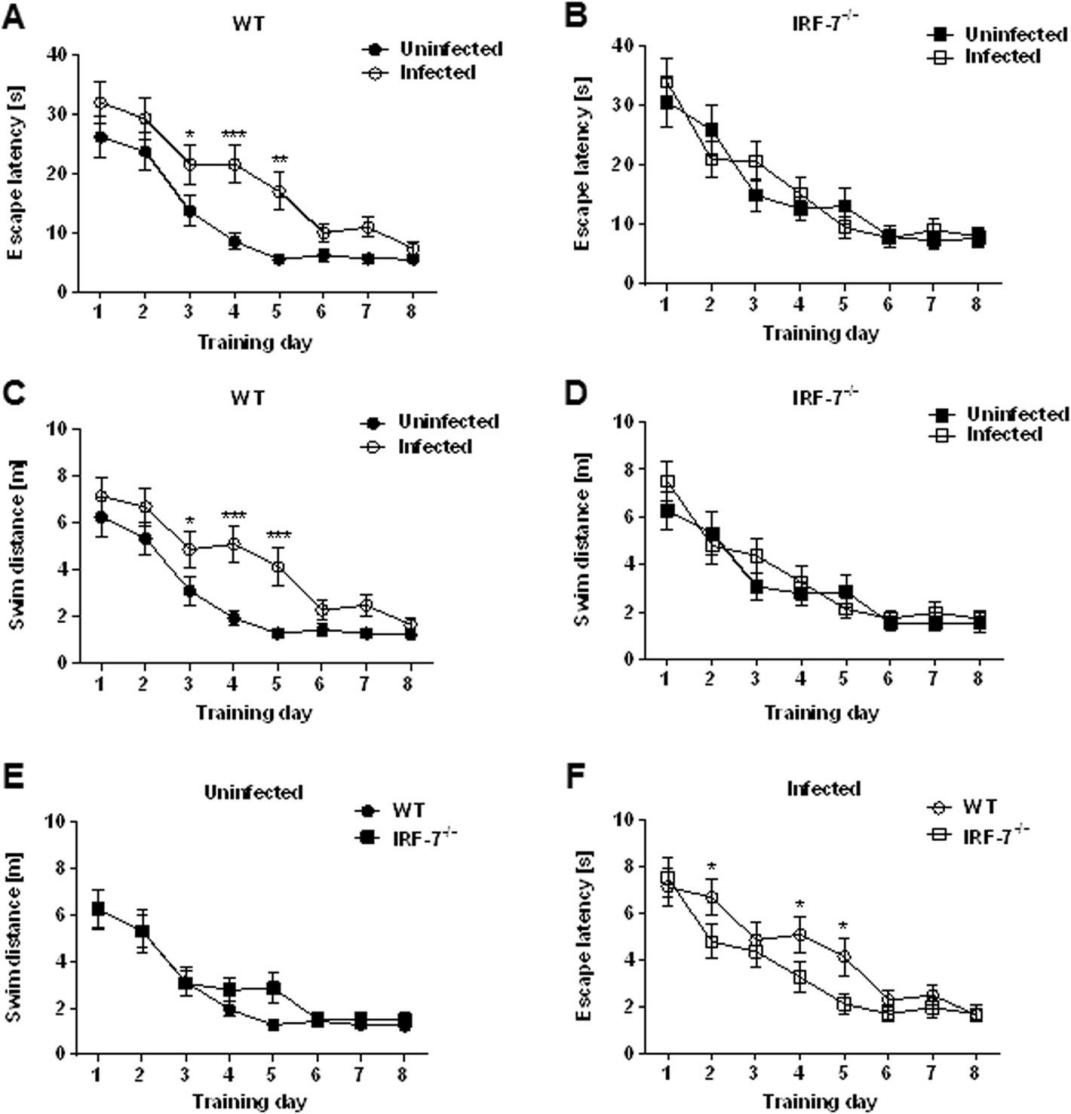
LGTV infection led to cognitive impairment in wildtype mice. Uninfected control (WT: *n* = 9, *Irf-7*^*−/−*^: *n* = 7) and LGTV-infected (10^4^ FFU, i.p.) mice (WT: *n* = 10, *Irf-7*^*−/−*^: *n* = 9) were trained in the Morris water maze test for 8 consecutive days. **a**, **b** Escape latency and **c**–**f** swim distance of control and infected wildtype and *Irf-7*^*−/−*^ mice are shown. Two-way ANOVA with one repeated measure and post hoc Student’s *t* test using Fisher’s least significant differences were employed to analyze the data. Data are shown as mean ± SEM, *p < 0.05, **p < 0.01, and ***p < 0.001

In the Morris water maze test, swimming time (escape latency) and path length (swim distance) to reach the platform can be used as measures for memory formation over consecutive days [26, 27]. During 8 days of acquisition training, the escape latency and swim distance to reach the platform declined and thereby indicated hippocampus-dependent spatial learning and memory formation in all tested groups, (Fig. 5) (escape latency: repeated measure one-way ANOVA: *F*_Uninfected WT_ (7, 56) = 14.24, *p* < 0.001; *F*_Infected WT_ (7, 63) = 8.38, *p* < 0.001; F_Uninfected *Irf-7−/−*_ (7, 42) = 6.87, *p* < 0.001; F_Infected *Irf-7−/−*_ (7, 56) = 6.18, *p* < 0.001; swim distance: repeated measure one-way ANOVA: *F*_Uninfected WT_ (7, 56) = 15.06, *p* < 0.001; *F*_Infected WT_ (7, 63) = 8.56, *p* < 0.001; *F*_Uninfected *IrfF-7−/−*_ (7, 42) = 8.32, *p* < 0.001; *F*_Infected *Irf-7−/−*_ (7, 56) = 6.58, *p* < 0.001). Yet, the escape latency (Fig. 5a) and swim distance (Fig. 5c) in LGTV-infected wildtype mice were increased on day 3, day 4, and day 5 of acquisition training in LGTV-infected WT mice as compared to uninfected wildtype mice (escape latency: day 3 *p* = 0.024; day 4 *p* = 0.0002, day 5 *p* = 0.001; swim distance day 3 *p* = 0.038; day 4 *p* = 0.0002, day 5 *p* = 0.0007). These results suggest an impairment in spatial learning and memory formation following LGTV infection in wildtype animals (escape latency—two-way RM ANOVA: *F*_Treatment_ (1, 74) = 29.40, *p* < 0.001; swim distance—two-way RM ANOVA: *F*_Treatment_ (1, 74) = 26.23, *p* < 0.001). In contrast, analysis of the escape latency (Fig. 5b) and swim distance (Fig. 5d) in *Irf-7*^*−/−*^ mice did not reveal any significant differences (escape latency: two-way RM ANOVA: *F*_Treatment_ (1, 62) = 0.18, *p* = 0.66; swim distance: two-way RM ANOVA: *F*_Treatment_ (1, 62) = 1.19, p = 0.27). Furthermore comparison of the swim distance to reach the platform between wildtype and *Irf-7*^*−/−*^ uninfected control mice did not show any significant changes (Fig. 5e) (swim distance—two-way RM ANOVA: *F*_Treatment_ (1, 62) = 1.81, *p* = 0.18). However, LGTV infection in mice led to an increased swim distance in wildtype mice as compared to *Irf-7*^*−/−*^ mice (Fig. 5f) (swim distance—two-way RM ANOVA: *F*_Treatment_ (1, 74) = 7.26, *p* = 0.008).

**Fig. 6:**
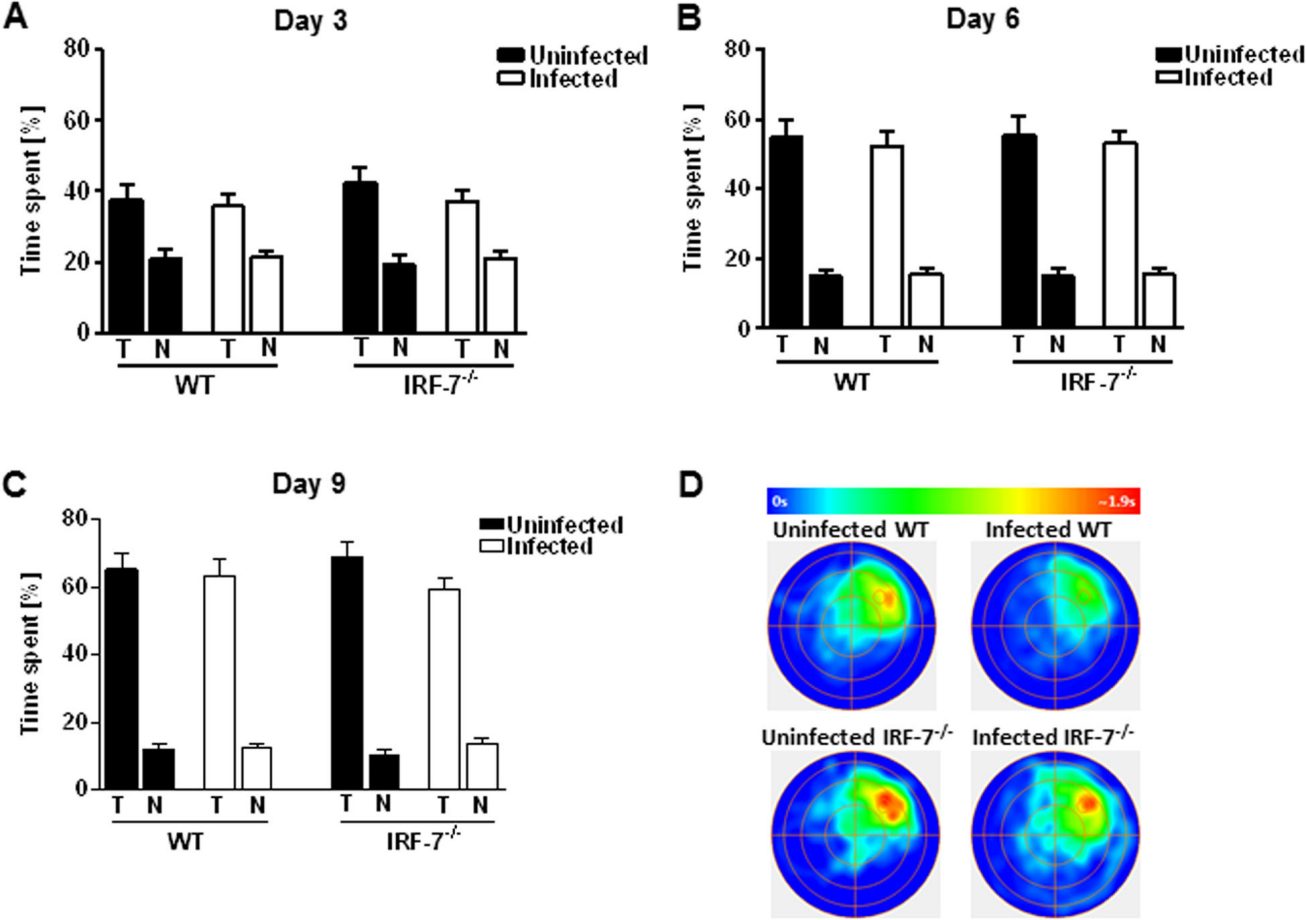
Memory retrieval was not affected following LGTV infection. Memory retrieval was assessed using probe trial tests performed on days 3, 6, and 9 of the training period in the Morris water maze. **a**–**c** The percentage of time spent in the target quadrant (T, north east) in comparison with the average time spent in non-target quadrants (N) by control and LGTV-infected wildtype and *Irf-7*^*−/−*^ mice (*n* = 7–10) is shown. **d** Group mean heat maps of the animals’ center position are illustrated for all tested groups. One-way ANOVA and post hoc Student’s *t* test using Fisher’s least significant differences were employed to analyze the data. Data are shown as mean ± SEM

While the escape latency and swim distance provide a read-out for memory acquisition, the reference memory test (probe trial) provides quantification for the retrieval of the specific memory. The probe trial tests were performed at days 3, 6, and 9 before the actual training began (Fig. 6). The probe trial tests revealed that the percentage of time spent in the target quadrant (T) increased over the training time in all groups (WT uninfected—one-way RM ANOVA: *F*_Treatment_ (2, 16) = 9.007, *p* = 0.002; WT infected—one-way RM ANOVA: *F*_Treatment_ (2, 18) = 13.13, *p* < 0.001; *Irf-7*^*−/−*^uninfected—one-way RM ANOVA: *F*_Treatment_ (2,12) = 11.94, *p* = 0.001; *Irf-7*^*−/−*^ infected—one-way RM ANOVA: *F*_Treatment_ (2, 16) = 10.42, *p* = 0.001). The quadrant preference was comparable between all tested groups, regardless of the genotype and whether the mice had been infected (day 3—one-way ANOVA: *F*_Treatment_ (3, 31) = 0.47, *p* = 0.70, Fig. 6a; day 6—one-way ANOVA: *F*_Treatment_ (3, 31) = 0.10, *p* = 0.95, Fig. 6b; day 9—one-way ANOVA: *F*_Treatment_ (3, 31) = 0.69, *p* = 0.56, Fig. 6c). However, according to the heat maps of the animals’ center position, it seems that LGTV infection in wildtype mice caused less concentration for searching in the target area on probe trial day 9 (Fig. 6d).

**Fig. 7:**
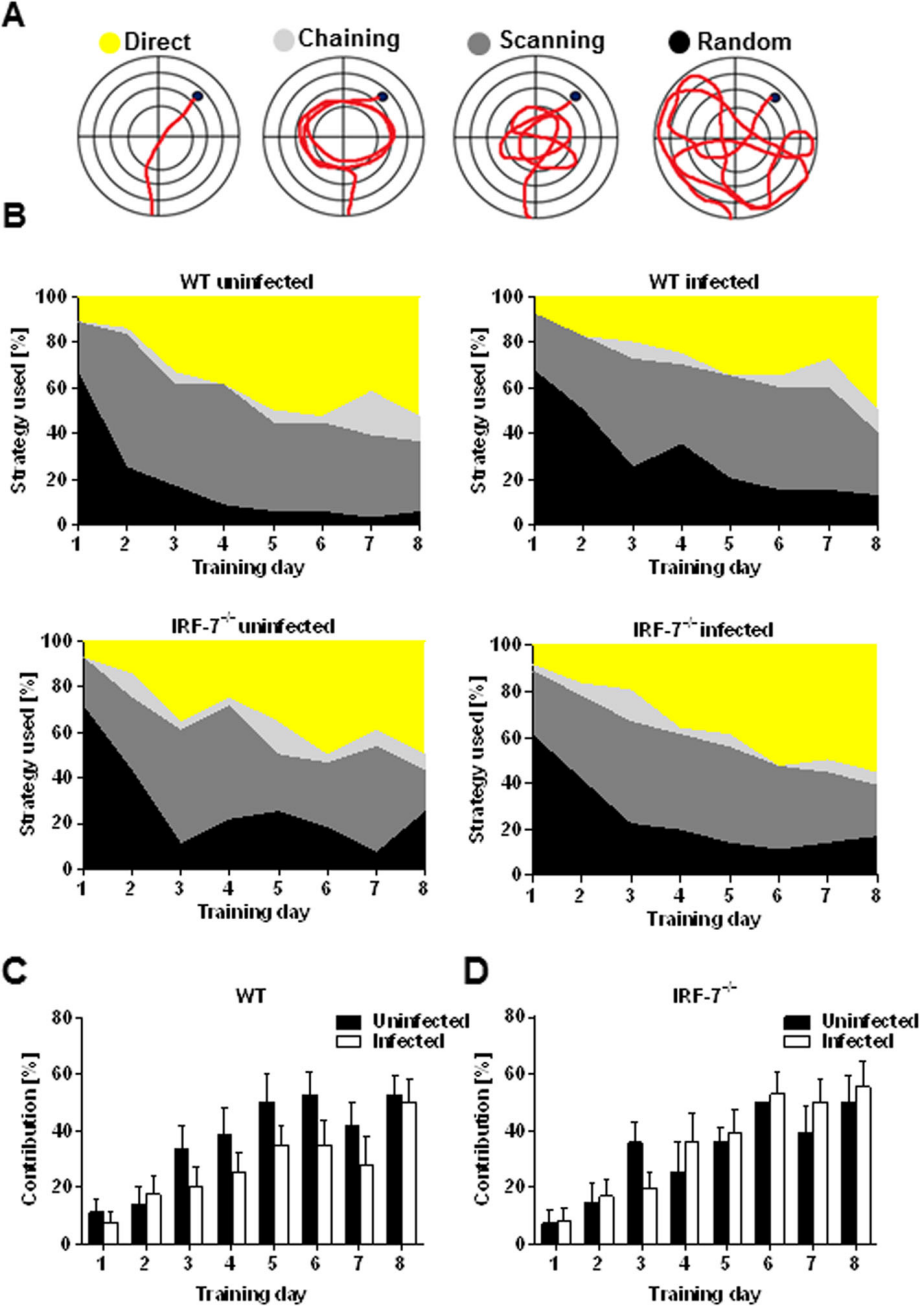
Analysis of learning strategies revealed a spatial learning impairment in LGTV infected wildtype mice. **a**, **b** Different searching strategies to locate the hidden platform during the 8 days of acquisition training in the Morris water maze test were assessed. **c**–**d** The augmentation of the hippocampus-dependent search strategy (directed search) in control and infected wildtype and *Irf-7*^*−/−*^ mice (*n* = 7–10) is shown. Two-way ANOVA with one repeated measure and post hoc Student’s *t*-test using Fisher’s least significant differences was employed to analyze the data. Data are shown as mean ± SEM

A detailed analysis of the swimming path allows for a qualitative assessment of learning in mice. Over time, healthy animals progressively switch from egocentric (hippocampus-independent: chaining, scanning, and random swimming) to allocentric (hippocampus-dependent: directed search) strategies to navigate to the hidden platform while a spatial map of the maze is formed (Fig. 7a) [29, 30]. All groups of control and LGTV-infected mice showed an augmentation of the hippocampus-dependent searching strategy during the 8 days of training (Fig. 7b). However, this progression seemed to be decreased for LGTV-infected wildtype mice compared to wildtype control mice (Fig. 7c) (two-way RM ANOVA: *F*_WT_ (1, 17) = 4.49, *p* = 0.04). No significant differences were detectable in the relative percentage of hippocampus-dependent strategy used between *Irf-7*^*−/−*^ LGTV-infected and control mice (Fig. 7d) (two-way RM ANOVA: *F*_*Irf-7−/−*_ (1, 14) = 0.50, *p* = 0.48). Thus, LGTV infection impacts hippocampus-dependent searching strategies only in WT mice.

**Fig. 8:**
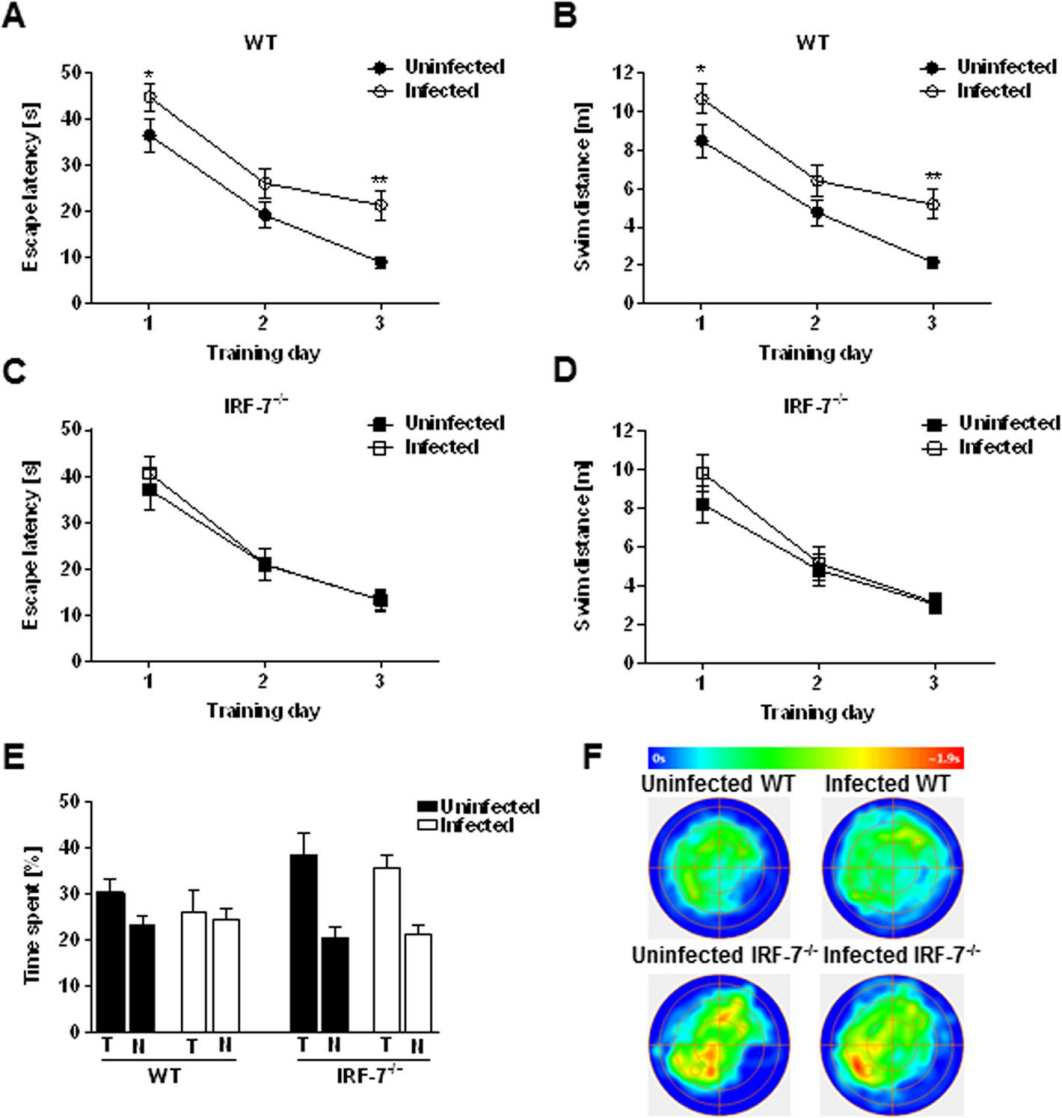
LGTV infection impaired memory formation for a new platform position in wildtype mice. Uninfected control (WT: *n* = 9, *Irf-7*^*−/−*^: *n* = 7) and LGTV-infected (10^4^ FFU, i.p.) mice (WT: *n* = 10, *Irf-7*^*−/−*^: *n* = 9) were trained for another 3 consecutive days of reversal training in the Morris water maze following initial acquisition. **a**, **b** The escape latency and swim distance to reach a new position of the hidden platform (SW) in wildtype and **c**, **d** *Irf-7*^*−/−*^ mice are presented. **e** A single probe trial test 24 h after the last day of reversal training was shown. The percentage of time spent in the target quadrant (T, south west) in comparison with the average time spent in non-target quadrants (N) by control and LGTV infected wildtype and *Irf-7*^*−/−*^ mice are shown. **f** Group mean heat map of the animals’ center position for all tested groups are illustrated. Two-way ANOVA with one repeated measure and post hoc Student’s *t* test using Fisher’s least significant differences was employed to analyze the data. Data are shown as mean ± SEM, *p < 0.05 and **p < 0.01

Once a spatial map including the distal cues is formed, a new platform position only need to be updated in the already existing cognitive map and can therefore be memorized faster. However, depending on the cognitive flexibility of the animal, the two memories can also compete with each other, which can be seen especially during the probe trial test [20]. In the reversed Morris water maze paradigm, the hidden platform was moved to the opposite quadrant (south west). During 3 days of training, the escape latency and swim distance to reach the new platform position decreased in all tested groups (Fig. 8a–d) (escape latency: repeated measure one-way ANOVA: *F*_Uninfected WT_ (2, 16) = 44.95, *p* < 0.001; *F*_Infected WT_ (2, 18) = 18.65, *p* < 0.001; *F*_Uninfected *Irf-7−/−*_ (2, 12) = 14.36, *p* = 0.001; F_Infected *IrfF-7−/−*_ (2, 16) = 19.64, *p* < 0.001; swim distance: repeated measure one-way ANOVA: *F*_Uninfected WT_ (2, 16) = 48.22, *p* < 0.001; *F*_Infected WT_ (2, 18) = 16.44, *p* < 0.001; *F*_Uninfected *Irf-7−/−*_ (2, 12) = 12.79, *p* = 0.001; F_Infected *Irf-7−/−*_ (2, 16) = 19.11, *p* < 0.001). However, LGTV-infected wildtype mice showed an increased escape latency (Fig. 8a) and swim distance (Fig. 8b) as compared to uninfected controls (escape latency: two-way RM ANOVA: *F*_Treatment_ (1, 74) = 12.15, *p* = 0.0008; swim distance: two-way RM ANOVA: *F*_Treatment_ (1, 74) = 12.42, *p* = 0.0007). No significant differences in escape latency (Fig. 8c) and swim distance (Fig. 8d) were detectable between LGTV infected and uninfected *Irf-7*^*−/−*^ mice (escape latency: two-way RM ANOVA: *F*_Treatment_ (1, 62) = 0.14, *p* = 0.70; swim distance two-way RM ANOVA: *F*_Treatment_ (1, 62) = 0.86, *p* = 0.35). Therefore, LGTV infection led to a reduced ability to memorize the new location of the hidden platform only in wildtype mice.

Subsequently, a single probe trial test 24 h after the last day of reversal training was performed (Fig. 8e, f). Uninfected wildtype, uninfected *Irf-7*^*−/−*^ and LGTV-infected *Irf-7*^*−/−*^ mice spent more time in the new target quadrant (T, south-west) as compared to the average time spent in the non-target quadrants (N, *p* < 0.05). Whereas in LGTV-infected wildtype animals (*p* = 0.73), no preference for the new target quadrant could be observed (Fig. 8e, f). Consequently, LGTV infection impaired initial and reversal learning in the Morris water maze; however, the phenotype was compensated in *Irf-7*^*−/−*^ mice.

### Hippocampal neuron morphology

**Fig. 9:**
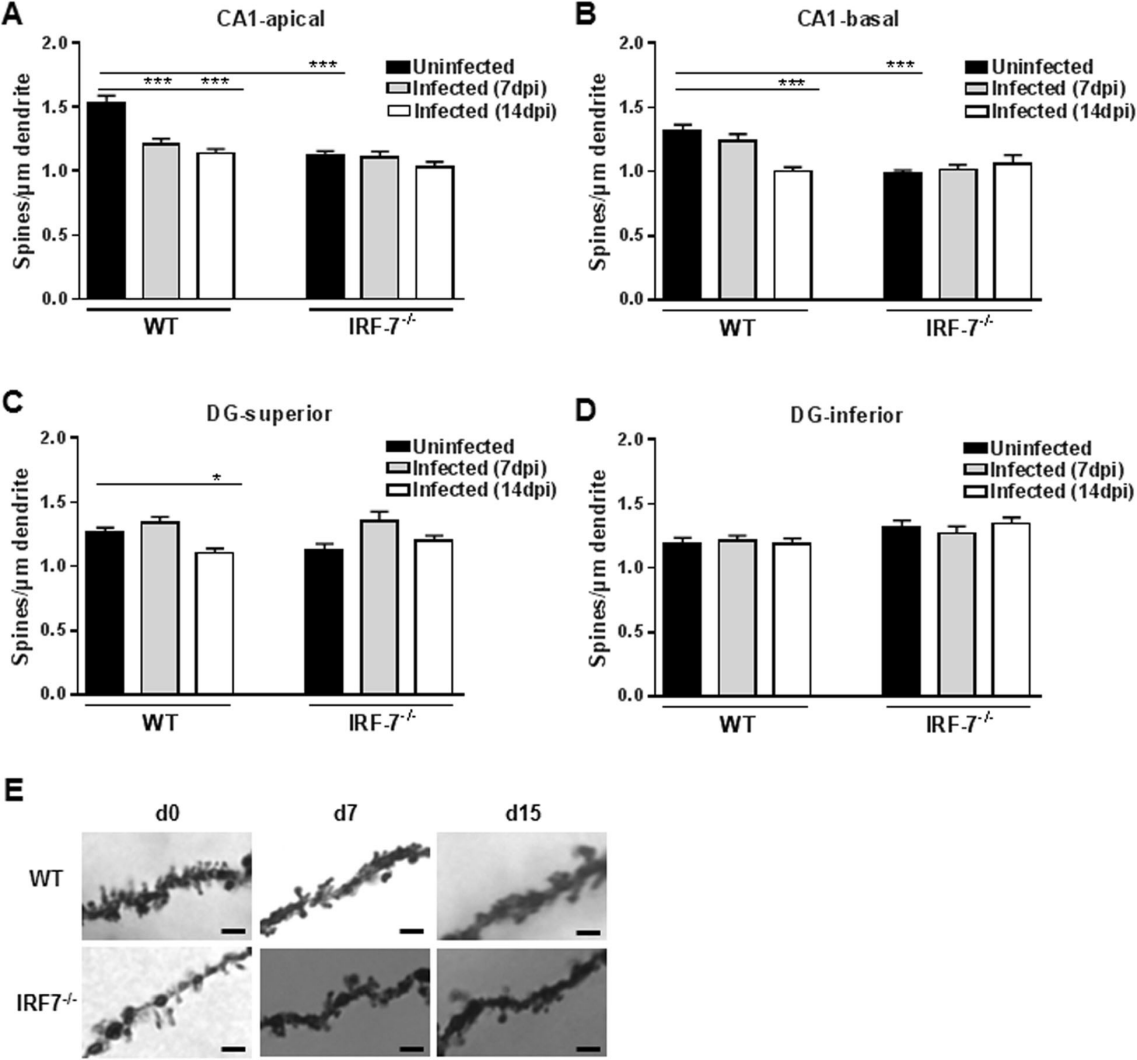
LGTV infection led to hippocampal spine density reduction in wildtype mice. Golgi-Cox staining was performed in the hippocampus of uninfected and LGTV-infected (10^4^ FFU, i.p.) mice after 7 and 14 days of infection. The dendritic spine density in **a** apical and **b** basal dendrites of hippocampal CA1 neurons and DG neurons located in **c** superior and **d** inferior were quantified (*n* = 3, number of dendrites in each group = 30–40). One-way ANOVA and post hoc Student’s *t* test using Fisher’s least significant differences were employed to analyze the data. Data are shown as mean ± SEM, *p < 0.05 and ***p < 0.001. **e** Representative pictures of Golgi-Cox staining of apical dendrites of CA1 neurons. Scale bar 2 μm

To investigate the potential cellular basis underlying the observed behavioral changes, hippocampal neuronal morphology was analyzed in uninfected and infected wildtype and *Irf-7*^*−/−*^ animals. Spines are tiny, dendritic protrusions that carry the majority of excitatory synapses in the hippocampus, and changes in spine density can provide information about alterations in the connectivity of hippocampal subregions [33]. Using Golgi-cox staining, spines were counted separately on apical and basal dendrites of CA1 and dentate granule cells located in the superior and inferior blade of the granule cell layer in the hippocampus (Fig. 8). In wildtype mice, the spine density in the apical and basal dendrites of CA1 pyramidal neurons decreased upon infection with LGTV (Fig. 9a, b). In the apical region, a significant spine reduction was detectable on day 7 and day 14 post-infection (Fig. 9a) (d7: Δ 23.14%, *p* < 0.001; d14 Δ 29.34%, *F* (2, 90) = 18.76, *p* < 0.001), whereas in the basal region, a significant dendritic spine reduction was detectable only 14 days post LGTV infection (Fig. 9b) (Δ 27.34%, *F* (2, 85) = 16.34, *p* < 0.001).

In addition, LGTV infection led to dendritic spine reduction in DG neurons located in the superior blade 14 days post-infection (Fig. 9c) (Δ 13.76%, *F* (2, 92) = 7.81, *p* = 0.02). Interestingly, infection with LGTV had no effects on dendritic spine numbers of CA1 and DG hippocampal subregions in *Irf-7*^*−/−*^ mice (Fig. 9a–d) (CA1: apical: *F* (2, 95) = 1.44, *p* = 0.24, basal: *F* (2, 84) = 0.67, *p* = 0.51; DG: superior: *F* (2, 91) = 0.94, *p* = 0.22, inferior: *F* (2, 88) = 0.57, *p* = 0.56). Taken together, LGTV infection reduced the spine density in the CA1 region in wildtype mice. *Irf-7*^*−/−*^ mice have a low spine density, which is not influenced by the LGTV infection.

### Microglia and astrocyte density and activity

**Fig. 10:**
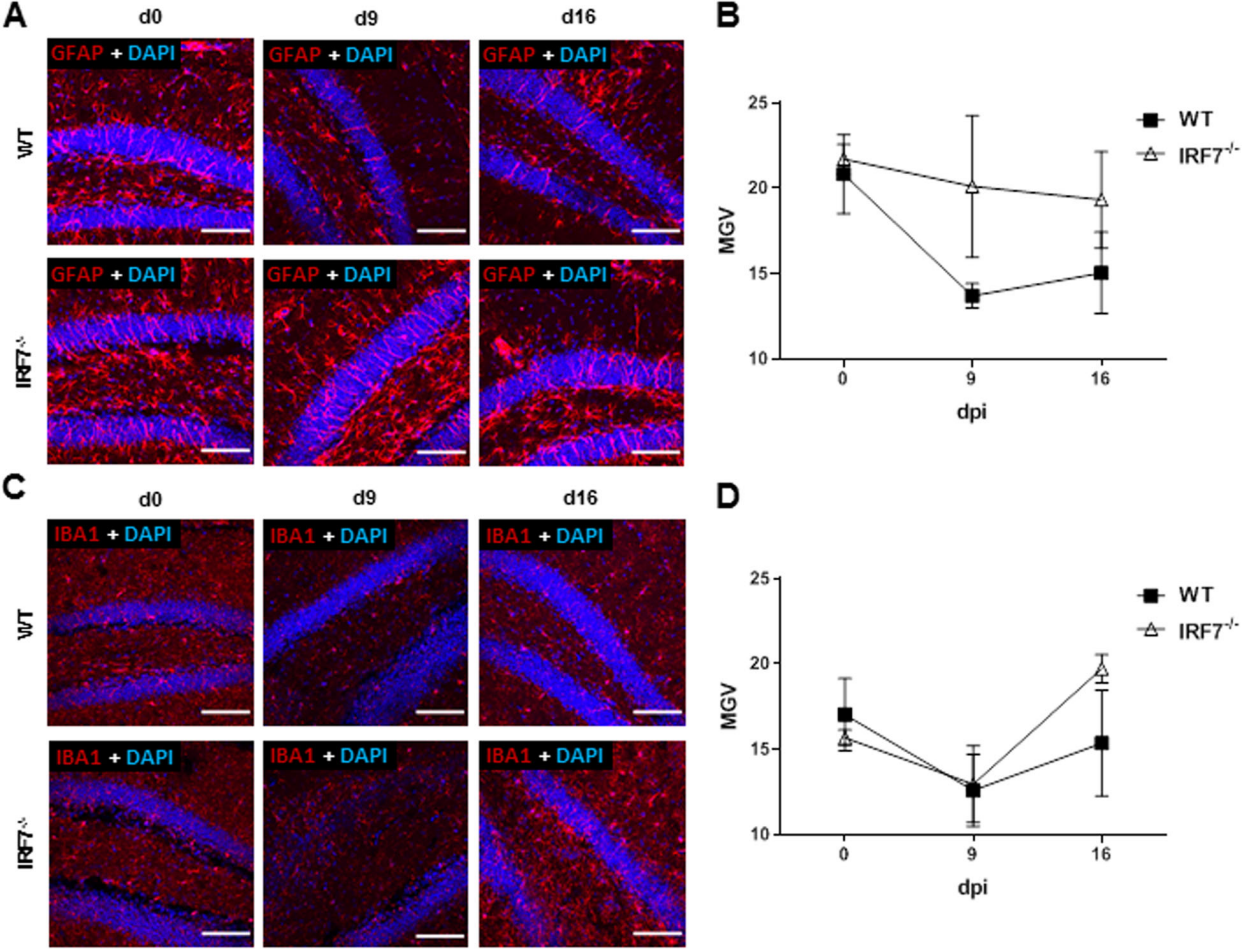
Glia cell activation in the hippocampus. Wildtype and *Irf-7*^*−/−*^ mice were infected intraperitoneally with 10^4^ FFU of LGTV; brains were isolated for immunohistology. Depicted are immunohistological analyses of the hippocampus on 0, 9, and 16 days post-infection as a representative image from at least three mice per group, **a** GFAP (red) or **b** IBA1 (red), DAPI (blue). Magnification × 20, scale bar 100 μm. Quantification of **c** GFAP and **d** IBA1 by mean gray value (MGV)

Since LGTV infection leads to alteration in spine density and behavior, we analyzed hippocampal glia cells for neuroinflammatory processes. The density of astrocytes in the hippocampus was analyzed using GFAP staining (Fig. 10a). Astrocyte density was decreased in the hippocampus of wildtype mice during the course of infection. At 16 days post-infection, when the virus was cleared, the number of activated astrocytes increased (Fig. 9b). In contrast, the infection had no dramatic impact on the activation status of the astrocytes in *Irf-7*^*−/−*^ mice (Fig. 10a, b).

For microglia cells, infection with LGTV decreased the overall density of activated IBA1^+^ cells 9 days post-infection (Fig. 10c, d). In contrast, the number of activated cells increased on day 16 post-infection to higher levels than before infection. These data indicate that LGTV infection in wildtype and *Irf-7*^*−/−*^ mice influence the activation of glia cells, although direct viral replication in the hippocampus was hardly detectable.

## Discussion

Severe infections with TBEV can lead to long-lasting health problems. However, two-thirds of human TBEV infections are non-symptomatic. Less is known about the impact of non-symptomatic infections on anxiety and memory formation. We therefore investigated whether infections without signs of disease directly affected the brain and behavior. We compared LGTV infection in wildtype mice, showing a very low viral replication in the olfactory bulb but no disease signs, with susceptible IRF-7 deficient mice, in which the virus replicates to higher titers in the hippocampus and the animals experience weight loss and mild signs of discomfort but eventually recover. The infection in wildtype mice induced impairments in cognitive functions associated with changes in the hippocampal morphology and synaptic properties of the neurons.

Here, we showed that spatial memory for the hidden platform test was formed irrespectively of the genetic strain and infection status. Infected wildtype mice performed worse than uninfected controls in the intermediate phase of the training period and used more hippocampus-independent search strategies (Fig. 7).

Inapparently, LGTV-infected wildtype mice showed less anxiety in the open field test compared to uninfected mice. The amygdala is a central area of processing fear and anxiety [34]. Although viral infection by human herpes virus 6 (HHV6) could lead to significant damage to the medial temporal lobe including the amygdala [35], we were not able to detect pathological alterations in the hippocampus upon LGTV infection [8]. Therefore, alternative mechanisms not associated to direct brain damage must be responsible. One possible mechanism is an indirect activation of brain regions, which has been shown for anxiety regulation during gastrointestinal infections [36].

Virus-induced behavioral alterations in mice have been previously reported for different viruses. Influenza virus infections have also led to impairments in cognitive functions during the acute phase of symptomatic infections [37]. Other studies show that this effect was not limited to acute infection and lasts longer [20]. For WNV, another neurotropic flavivirus, infection led to spatial learning and memory impairments [38]. However, these effects were determined after high viral replication in the CNS, which is in contrast to the low viral replication present in wildtype mice upon LGTV infection.

Alterations in the spine density of the hippocampus could lead to changes in memory formation [39, 40]. We analyzed the spine density of pyramidal neurons in the CA1 and DG region of the hippocampus. LGTV infection led to a reduction of spines in the CA1 neurons. A similar effect could be observed for WNV infection, where spine loss was mainly detected in the CA3 regions, whereas other regions were less affected [41]. This suggests that spine loss induced by virus infections in the hippocampus could be a possible mechanism of cognitive dysfunction. Interestingly, uninfected *Irf-7*^*−/−*^ mice showed less spine numbers of CA1 neurons than uninfected wildtype controls (Fig. 9a–d).

Proinflammatory processes play a role in stress-increased anxiety [42, 43], and the hippocampus is vulnerable to inflammation. Inflammatory responses could be induced in the brain, either by direct viral replication in the hippocampus, across multiple brain regions, or by peripheral inflammations. Inflammatory mediators produced during infection, affected neuronal morphology, synaptic structure, and function [12, 44]. In vivo stimulation with poly I:C, LPS, or different cytokines can modulate learning, memory formation, and synaptic plasticity [45–47], and LGTV infection leads to the induction of proinflammatory cytokines in WT mice in the olfactory bulb, but not directly in the hippocampus (Fig. 2c). However, effects in trans could not be excluded.

*Irf-7*^*−/−*^ is a major regulator of the type I IFN system. The effect of type I IFN in the brain is stated to be versatile and is associated with attenuation of neuroinflammation and protection from neurodegeneration [48–51] or contributes to neuronal aging [52, 53]. This indicates a differential role of the type I IFN system in the development, homeostasis, and aging of the brain. In our study, *Irf-7*^*−/−*^mice displayed a lower number of spines in CA1 neurons as compared to wildtype mice in general. As these mice are conventional knock-out mice, and they are born with *Irf-7* deficiency, compensatory mechanisms possibly occur during development. Interestingly, no alteration in spine density was detectable in any region of the hippocampus in *Irf-7*^*−/−*^ mice upon infection, which is in line with the observation that a LGTV infection showed no cognitive impairment for this genotype. *Irf-7*^*−/−*^ mice had lower spine numbers compared to WT mice. Reduced spine numbers were also observed in *Ifnar-1*^*−/−*^ deficient mice [54]. Since IRF-7 is a prominent regulator of the type I IFN system, it could be involved in the regulation of synaptic plasticity. As spine numbers were not changed by virus infection in *Irf-7*^*−/−*^ mice, cognitive impairment seems to be independent of the overall spine number but on the relative number of spines. Moreover, due to higher viral replication and inflammatory responses in the *Irf-7*^*−/−*^ mice, spines seem to be less sensitive. The underlying mechanism remains elusive and needs to be characterized in detail in uninfected *Irf-7*^*−/−*^ mice.

Microglia are required for environment-induced brain plasticity and for learning-induced synapse formation [55, 56] and activation of microglia could be associated with the shaping of spines from hippocampal neurons. Virus infection led to the activation of microglia in infected wildtype and *Irf-7*^*−/−*^ mice (Fig. 10b). Microglia activation is regulated by switching the cells from M1 proinflammatory to an M2 tissue regenerative phenotype after the acute phase of infection. IRF-7 is responsible for this M1 to M2 switch in microglia [50]. Thus, retaining microglia in a proinflammatory M1 phenotype could be responsible for ongoing shaping of neuronal spines. A detailed analysis of the temporal course of microglia activation could bring further evidence for such a hypothesis. Microglia and astrocytes interact in formation and elimination of synaptic connection [57, 58], and in WNV infection, T cells promoted microglia-mediated synaptic elimination and cognitive dysfunction [59].

The number of activated astrocytes increased upon infection in both wildtype and *Irf-7*^*−/−*^ mice. During WNV virus replication in the CNS, proinflammatory astrocytes impaired neuronal progenitor cell homeostasis via expression of IL-1 [41]. However, whether astrocytes differed in their cytokine expression profile during infection with or without signs of disease is unclear and need further investigation.

Taken together, our findings indicate that a CNS infection without signs of disease can lead to impairments in cognitive functions through local inflammatory responses in the hippocampus. Since chronic neuroinflammation or repeated virus reactivation is associated with neurodegenerative diseases [60], mild infections of the CNS could display relevant risk factors.

## Conclusion

**Fig. 11:**
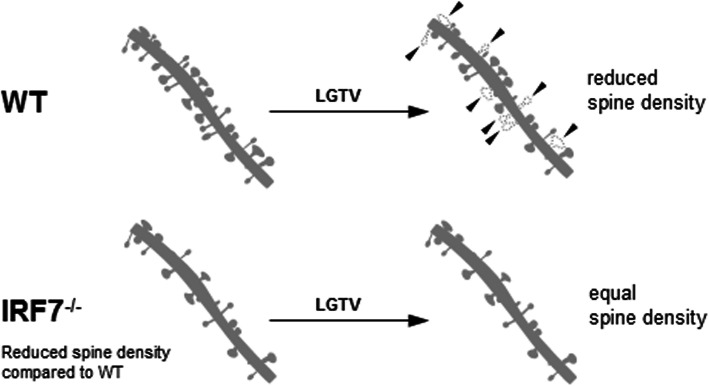
Graphical summary. LGTV infection leads to the reduction of spine density in apical and basal dendrites of CA1 pyramidal neurons 14 days post-infection. The number of spines is reduced in uninfected *Irf-7*^*−/−*^ mice compared to WT mice. LGTV infection does not change spine density in *Irf-7*^*−/−*^ mice. Spines were not classified by morpholgy

The present study investigated the impact of LGTV infection with or without signs of disease on spatial memory. By performing different behavior- and memory-related tests, we observed a significant decrease of anxiety and changes in memory formation after infection. The local infection of the olfactory bulb mediated far-reaching effects in the hippocampus and led to changes in the spine density of CA1 neurons (Fig. 11) and the frequency of activated microglia and astrocytes. Since the number of spines in uninfected *Irf-7*^*−/−*^ mice was reduced, we suggested that the type I IFN system play a considerable role in the regulation of brain morphology and function. In summary, our data show that LGTV infection with no or only mild signs of disease seems to influence the regulation of anxiety and memory. Future studies should be focused on elucidating the underlying mechanisms.

## Data Availability

The datasets used during the current study are available from the corresponding author on reasonable request.

## Abbreviations

TBEV: Tick-borne encephalitis virus
TBE: Tick-borne encephalitis
IRF-7: Interferon regulatory factor-7
LGTV: Langat virus
DENV: Dengue virus
JEV: Japanese encephalitis virus
WNV: West Nile virus
YFV: Yellow fever virus
ZIKV: Zika virus
CNS: Central nervous system
IFN: Type I interferon
IL-1β: Interleukin-1β
IL-6: Interleukin-6
TNF-α: Tumor necrosis factor-α
DG: Dentate gyrus
CA: Cornu Ammonis
LTP: Long-term potentiation
WT: Wild type
i.p.: Intraperitoneal
FFU: Focus formation unit
MWM: Morris water maze
